# Dispersal and mating patterns determine the fate of naturally dispersed populations: evidence from *Bombina orientalis*

**DOI:** 10.1186/s12862-021-01844-3

**Published:** 2021-06-07

**Authors:** Liqun Yu, Shuai Zhao, Fanbing Meng, Yanshuang Shi, Chunzhu Xu

**Affiliations:** College of Life Science, Northeast Agricultural University, No. 600 Changjiang Road Xiangfang District, Harbin, 150030 China

**Keywords:** *Bombina orientalis*, Genetic diversity, Mating system, Naturally dispersed population, Sex-biased dispersal

## Abstract

**Background:**

In contrast to the explosive increase of a population following biological invasion, natural dispersal, i.e., when a population disperses from its original range into a new range, is a passive process that is affected by resources, the environment, and other factors. Natural dispersal is also negatively impacted by genetic drift and the founder effect. Although the fates of naturally dispersed populations are unknown, they can adapt evolutionarily over time to the new environment. Can naturally dispersed populations evolve beneficial adaptive strategies to offset these negative effects to maintain their population in a stable state?

**Results:**

The current study addressed this question by focusing on the toad *Bombina orientalis*, the population of which underwent natural dispersal following the Last Glacial Maximum in Northeast Asia. Population genetic approaches were used to determine the genetic structure, dispersal pattern, and mating system of the population of *B. orientalis* in northeast China (Northern population). The results showed that this northern population of *B. orientalis* is a typical naturally dispersed population, in which the stable genetic structure and high level of genetic diversity of the population have been maintained through the long-distance biased dispersal behavior of males and the pattern of promiscuity within the population.

**Conclusions:**

Our findings suggest that naturally dispersed populations can evolve effective adaptive strategies to maintain a stable population. Different species may have different strategies. The relevance of these maintenance mechanisms for naturally dispersed populations provide a new perspective for further understanding the processes of speciation and evolution.

**Supplementary Information:**

The online version contains supplementary material available at 10.1186/s12862-021-01844-3.

## Background

The expansion of a population from its original habitat to a new area forms the basis for the development of, and changes in, phylogeographic patterns and biological dispersal behavior. Natural dispersal usually takes a significant period of time, and stable or even differentiated populations might form. However, in other cases, the naturally dispersed population might gradually reduce and disappear in fluctuating ecosystems. This uncertainty has aroused the interest of researchers to explore the fate of naturally dispersed populations [1]. By contrast, the ecological effect of redispersal behavior after biological invasion is easier to determine and has become a research focus in both ethology and ecology. For example, Wang et al. (2018) revealed the taxonomic diversity indices of plant communities significantly decreased under moderate and heavy degree of *Solidago canadensis* invasion conditions [2]. In another research, the existing geographical distribution of invasive cane toads (*Rhinella marina*) which formed after the redispersal in Australia was consistent with the central marginal hypothesis predictions, that is, the genetic diversity of the marginal population was lower than that of the central population [3]. Biedrzycka et al. (2014) inferred invasion routes through the study of the existing genetic structure of species [4]. It has been proposed that biological invasion and natural dispersal are essentially the same [5], although we consider them to be very different. Most invasive populations result from human-mediated extra-range dispersal events or other factors and spread rapidly after invading the new area, forming a population with a stable genetic structure in a short time frame. By contrast, natural dispersal is usually achieved by the gradual spread of a population from its original to a new range, or through a suitable habitat corridor. The natural dispersal of a population is a complex process, which is affected by resource competition and environmental change, and the time between the occurrence of dispersal behavior to the formation of a new species distribution pattern is significant [6]. In addition, such dispersal provides a prerequisite for the evolution of adaptive traits (e.g. behavioral phenotypes). Different from the rapid expansion of population after the biological invasion, if the naturally dispersed population need to form a stable geographical pattern, it will inevitably experience genetic drift, the founder effect, inbreeding depression and many other evolutionary processes that may lead to population decline [7, 8]. Then, can the naturally dispersed population evolve adaptive strategies that offset these negative effects to maintain a stable or even differentiated population?

The Oriental fire-bellied toad (*Bombina orientalis*) is an amphibian that is distributed across Northeast Asia [9]. The population of *B. orientalis* has been studied using mitochondria markers, and was found to have a stable genetic structure [10]. The northern population (northeastern China population) originated from the Korean Peninsula refuge (peninsular population) via natural dispersal processes after the Last Glacial Maximum (LGM, ~ 22,000 years ago) of the Quaternary [10]. The Korean Peninsula was a biological refuge in Northeast Asia during the Quaternary glaciation [11, 12]. However, as a naturally dispersed population, it is unclear what determined the fate of the northern *B. orientalis* population. Shi et al. (2018) did basic analysis on genetic diversity of the northern population while developing microsatellite markers and found high level genetic diversity [13]. In this study, population genetics methods were used to explore whether the dispersal and mating patterns of *B. orientalis* population are beneficial to maintaining population stability.

## Results

### Population genetic diversity

**Fig. 1:**
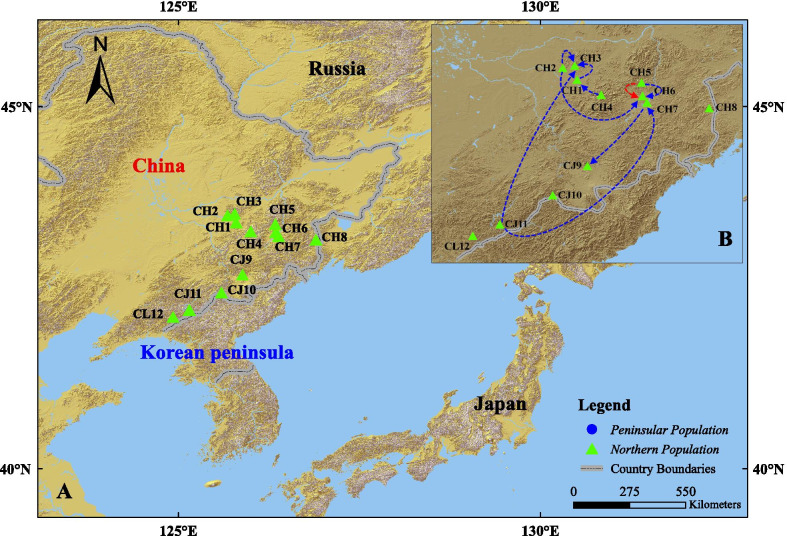
**A** Map of the sampling sites in Northeast Asia. Detailed sampling site information is also presented in Additional file 1: Table S1.  CH, Heilongjiang province of China; CJ, Jilin province of China; CL, Liaoning province of China; 1, Chenjia Village; 2, Xiaoling; 3, Maoer Mountain; 4, Weihe; 5, Weihu Mountain; 6, Sandaoguan; 7, Mudanfeng; 8, Dongning; 9, Lushui River; 10, Linjiang; 11, Yulin Town; 12, Kuandian. **B** Identified migration routes of males (blue-dashed lines) and females (red-dashed lines). Each line represents a dispersed genotype that was found in a place far away from their birth-place

131 samples from the northern population were all successfully amplified and sequenced in this study (GenBank accession numbers: MK609566–MK609843). The peninsular population included 127 samples that were retrieved from GenBank [10] (GenBank accession numbers: KR869225–KR869512). In total, 258 concatenated mtDNA *COI* (903 bp) and *Cytb* (885 bp) samples were used for genetic diversity analysis. The results from the northern population were as follows: *H* = 38, *Hd* = 0.796 ± 0.035, *π* = 0.00189 ± 0.00014; whereas those of the peninsular population were: *H* = 95, *Hd* = 0.994 ± 0.002, *π* = 0.0192 ± 0.0008. Thus, the haplotype diversity of the northern population was high, but slightly lower than that of the peninsular population. Figure 1 A and Additional file 1: Table S1 contain information for all sampling sites.

Microsatellite data from 12 loci and 515 individuals were used to test linkage disequilibrium (LD) and Hardy-Weinberg equilibrium (HWE). The results showed that there was no LD across any pair of loci. Loci 12 F, 13, 17, 10 F, 42, 53, and B14 conformed to HWE in most independent populations (results are detailed in Additional file 1: Table S2). Genetic diversity analysis of 515 individuals was conducted based on these seven loci and showed that: *Na* = 5.036 ± 0.327, *Ne* = 2.961 ± 0.176, *He* = 0.568 ± 0.024, and *Ho* = 0.573 ± 0.027. The genetic diversity of each sampling site is shown in Additional file 1: Table S3.

### Mantel test

The isolation-by-distance (IBD) analysis revealed a significant correlation between genetic distance and geographic distance (*P*_Mantel_ < 0.0001, correlation coefficient r = 0.510). A linear correlation best-fit line is presented in Additional file 1: Fig. S1.

### Population history analysis

**Fig. 2:**
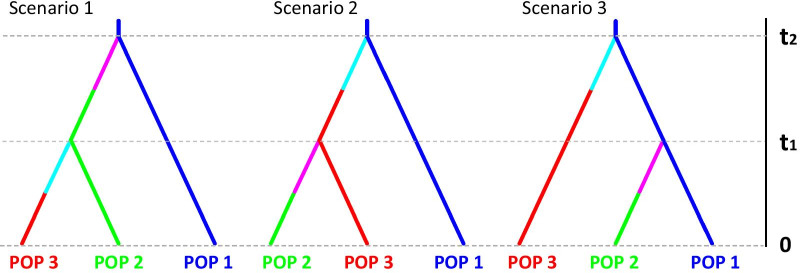
DIYABC scenarios tested on the various population groups. The following three scenario correspond to the classic dispersal history from an ancestral population (population 1). In scenario 1, population 3 is derived from population 2, which is derived from population 1. In scenario 2, population 2 is derived from population 3, which is derived from population 1. In scenario 3, both populations 2 and 3 are derived independently from population 1. Different color after the split of each population means a new population is created from its ancestral

DIYABC analyses between the northern population and the peninsula population showed that the posterior probability of Scenario 2 was highest (logistic regression = 0.5048, confidence intervals 0.4779–0.5316). The results of the logistic regression and confidence intervals of Scenario 1 and 3 are 0.4352 (0.4077–0.4627) and 0.0600 (0.0395–0.0806). These results indicate that population 2 (northern population in China) derived from population 3 (the northern group of peninsula population), which was derived from population 1 (the southern group of peninsula population). In the test of DIYABC within the northern population, the posterior probability of Scenario 1 was highest (logistic regression = 0.5792, confidence intervals 0.5267–0.6318), which indicated that the CJ9–CJ11, and CL12 populations first differentiated from the northern population. The logistic regression and confidence intervals of Scenario 2 and 3 are 0.2154 (0.1534–0.2774) and 0.2054 (0.0964–0.3143), respectively. The details of all such topologies are provided in Fig. 2. All of the above results support the hypothesis that the northern population of *B. orientalis* is a naturally dispersed population from the refugia of the Korean peninsula after LGM. By comparing the effective population sizes of the ancestral (*Nan*) and contemporary (*N*) populations, the results showed a significant increase in the northern population (Additional file 1: Table S4).

### Ecological niche modeling

Maxent predicted the highest habitat suitability during LGM for *B. orientalis* to be the southern Korean peninsula. From the Holocene to the present, the most suitable habitat for *B. orientalis* expanded to the northern, covering the current distribution range (Additional file 1: Fig. S2). This habitat suitability enabled the peninsular population to disperse northward and to form the current northern population.

#### Sex-biased dispersal

**Fig. 3:**
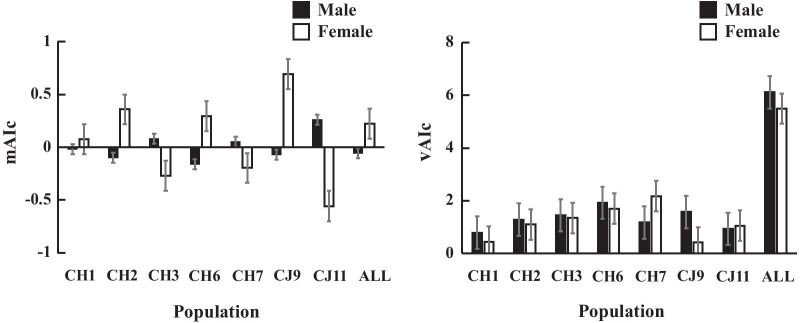
Comparison of the mean assignment index correlation (*mAIc*) and variance assignment index correlation (*vAIc*) between males and females

The corrected assignment index (*mAIc*) between males and females was calculated by using microsatellite data from the CH1, CH2, CH3, CH6, CH7, CJ9, and CJ11 populations and the whole northern population. The *mAIc* of males from the whole northern population (–0.054) was lower than that in females (0.223); whereas the *vAIc* of males (6.113) was higher than that of females (5.486) (Fig. 3). All these results were consistent with a male-biased dispersal hypothesis.

GENECLASS 2 identified similar proportions of first-generation migrants among males and females, although males had more long-distance dispersers compared with females. From 515 samples, ten males and two females were identified as first-generation migrants (Additional file 1: Table S5). The proportion of male migrants in the male subpopulation (10/400) was higher than that of females (2/103), but the difference was not statistically significant (Pearson’s chi-squared test, χ^2^ = 0.32, df = 1, *P* > 0.05). The mean dispersal distance was 154.19 km for males and 17.95 km for females (Fig. 1B and Additional file 1: Table S5). The average dispersal distance and frequencies of dispersal events at different distances for males were significantly higher than for females (Wilcoxon rank sum test, *P* < 0.001). These results are also consistent with a male-biased long-distance dispersal hypothesis.

The results of *F*_*ST*_ showed an *F*_*ST*_ value of nuclear DNA (0.1242) was lower than that of mtDNA (0.4266). The *F*_*ST*_ based on nuclear genes of males and females showed that males displayed lower genetic diversity (*F*_*ST*_ = 0.1212) than females (*F*_*ST*_ = 0.1312), again consistent with a male-biased dispersal hypothesis.

##### Mating system

*Bombina orientalis* exhibits sexual dimorphism and gender can be identified by appearance [9]. The male/female ratio was 400/103 in the whole northern population (Additional file 1: Table S6). Thus, it is clear that there is a male-biased sex ratio in this species.

**Fig. 4:**
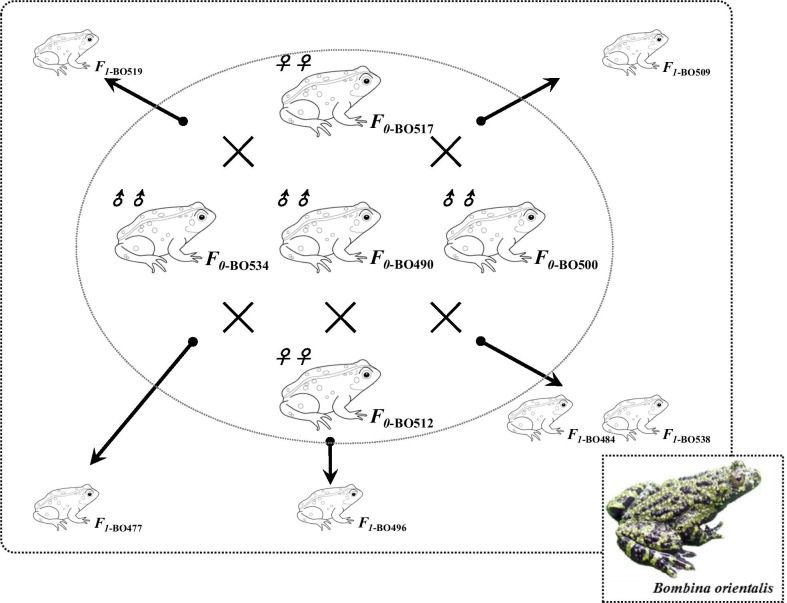
Promiscuity pattern diagram of *B. orientalis*. BO517 (♀) mates with BO534 (♂) and BO500 (♂) respectively, and the produced offspring are BO519 and BO509. BO512 (♀) mates with BO534 (♂), BO500 (♂) and BO490 (♂) respectively, and the produced offspring are BO477, BO484, BO538 and BO496. Identified multiple-mating individuals are presented in Additional file 1: Table S7

In total, 132 samples from the CH3 population and 12 microsatellite loci were used for parentage analysis in Cervus. Considering the 2017 result, at least six females mated with two males and produced different offspring, and three males mated with two females (Fig. 4). The results for three years (2016–2018) are shown in Additional file 1: Table S7, and indicate that *B. orientalis* has a promiscuous mating system.

The value of *F*_*IT*_ (total-population inbreeding coefficients, 0.1364) and *F*_*IS*_ (inbreeding coefficients, 0.1198) in the CH3 population were lower than those in the whole northern population (*F*_*IT*_ = 0.2402, *F*_*IS*_ = 0.1324).

## Discussion

### Characteristic differences between naturally dispersed and invasively dispersed populations

Biological glacial refuges and the theory of postglacial redispersal was one of the theories which can explain phylogeographic and biological diversity patterns in Northeast Asia [9, 11, 14]. Fong et al. focusing on the genetic structure of *B. orientalis* populations, reported that this is a representative species of amphibians in Northeast Asia, and that its northern population originated from the peninsula population [10]. In addition, through the analysis of ENM prediction, population genetic diversity, and evolutionary history, this study also verified that the northern *B. orientalis* population formed following natural dispersal from the peninsula population after the LGM. The ENM results showed that, since the LGM of the Quaternary, suitable habitat for *B. orientalis* populations gradually expanded from the Korean peninsula northwards, providing favorable conditions for the natural dispersal of *B. orientalis*. It also explains why the peninsula population has a very high level of genetic diversity (*Hd* = 0.994 ± 0.002, *π* = 0.0192 ± 0.0008). The genetic variation of a population reduces when it disperses from its original range to a new range [3, 15]. For example, climate change during the Quaternary glacial period caused the postglacial expansion of *B. orientalis* under natural conditions, with pioneer populations on the outer edge of this expansion showing reduced genetic variation [16]. Thus, the northern population of *B. orientalis* redispersed from the Korean peninsula (refuge) and colonized new areas after the LGM, indicating that this is a typical naturally dispersed population.

There are abundant researches on the driving mechanism and ecological effect of the re-dispersed behavior of invasive populations [17–19]. Genetic evidence of invasive populations combined with invasion historical records comprehensively showed that most biological invasion processes have multiple sources (including multiple locations, multiple populations, and multiple introductions), which is distinct from natural dispersal processes [20–22]. Of such processes, human-mediated dispersal tends to introduce higher levels of within-population genetic variation, is more likely to facilitate genetic admixture, and results in higher dispersal efficiency and better dispersal effects compared with natural dispersal, particularly for dispersal over large geographical distances [23, 24]. By contrast, marginal populations are more likely to be affected by genetic bottlenecks and founder effects during natural dispersal, with far-reaching consequences. Therefore, forming a stable population by natural dispersal requires a longer time frame compared with invasive redispersal, and will be impacted by the more complex temporal uncertainty of factors such as climate or human activity. Moreover, the redispersal of invasive populations from their new range to other new areas reflects an active dispersal process, forming a stable population in a short time frame. By contrast, the dispersal of naturally dispersed populations from their original to a new range, showing strongly philopatry habits, reflects natural dispersal and is successful only as a result of the adaptive evolution of traits over a longer time frame [22]. *Bombina orientalis* has experienced tens of thousands of years of adaptive dispersal and has evolved a beneficial dispersal pattern and mating system. Whether these traits can resist the influence of complex factors on the dispersal process to form natural colonization populations is an important area for future research to determine the successful colonization mechanisms of naturally dispersed population. It will also provide a scientific basis for revealing the maintaining mechanism of biological (genetic) diversity.

### Multiple factors rescued the fate of ***B. orientalis***

Biodiversity in Northeast Asia was significantly affected by the Quaternary glacial. Most species have experienced re-expansion from refuges after the glacial periods, and the level of genetic diversity of populations is generally low, and population sizes are unstable or even in decline [9, 11, 25]. However, the results of the current study showed that the *B. orientalis* population in northeast China has a higher level of genetic diversity compared with other amphibian species in Northeast Asia (*Hd* = 0.796 ± 0.035, *π* = 0.00189; *He* = 0.582 ± 0.071, *Ho* = 0.475 ± 0.071). For instance, the average genetic diversity of *Rana dybowskii* populations distributed in the same region is *He* = 0.572, *Ho* = 0.291 [26], and of *Rana nigromaculata* is *He* = 0.367 ± 0.038 and *Ho* = 0.342 ± 0.041 [27]. However, there was a degree of inbreeding (*F*_*IS*_=0.1324) in the *B. orientalis* population, although this was lower than that of other amphibians. For example, the average inbreeding coefficient of *R. dybowskii* populations in the same region was higher (*F*_*IS*_=0.504) [26]. The size of the *B. orientalis* population showed an increasing trend. For natural populations, genetic variation directly affects the fitness and evolutionary potential of the population [28], whereas inbreeding will reduce the genetic variation and increase the risk of extinction [29]. Combined with our analysis of genetic variation, it appears that the *B. orientalis* population has a beneficial way of performing gene exchange, whereby, although it cannot completely avoid inbreeding, it can effectively reduce the negative effects of inbreeding depression to maintain the stability of the population. This pattern of gene exchange results from the male-biased long-distance dispersal and promiscuity. A similar dispersal pattern exists in the fire salamander, where females appear to be more philopatric, whereas males show greater variation in dispersal distances [30]. Recently, increasing evidence shows that the personality traits of dispersed individuals and the environment are also factors that could help explain the causes of dispersal [31–33]. The stronger appendages of male *B. orientalis* might improve their migration ability, while their temporary breeding grounds experience rapid habitat changes, forcing *B. orientalis* to constantly look for new breeding grounds [34]; such dispersal behavior is a bet-hedging strategy in what is a temporary and unstable environment [35]. By spreading their progeny more evenly among different sites, genotypes with a higher dispersal ability are better able to sample habitat variation within a generation, thus reducing the generation-to-generation variance in their mean performance. Zajitschek et al. (2009) reported that the addition of male immigrants resulted in the highest levels of population growth, with the effect of male rescue being more obvious especially when there is inbreeding in the immigrant population [36]. Immigrant males could help to genetically recover population growth by potentially outcompeting inbred males in terms of sperm competition [36]. By contrast, the short-distance dispersal of females might be more beneficial to avoid inbreeding, and promiscuity could increase the utilization rate of germ cells [37]. The existence of migrating male individuals could also have a more cost-effective role in fertilization, producing more hybrid offspring with more females, improving the level of genetic variation, and enhancing the development potential of the population, laying the foundation for the future expansion of the population.

The male/female ratio is a crucial characteristic of any population because it is likely to affect competition for mates among individuals and, hence, the mating system, dispersal and migratory behavior, and demographics of the population [38–40]. Our field research found a significant male-biased sex ratio in the natural population of *B. orientalis* (♂/♀ = 400/103). Especially in breeding grounds, this provides a prerequisite for promiscuity, which is dominated by polyandrous individuals. Meanwhile, the competitive pressure of mating resources in the breeding grounds will also prompt males to emigration and dispersal [41]. The significant male-biased sex ratio of the *B. orientalis* population is mainly due to the increase in the number of males following egg hatching, a phenomenon caused by high temperatures during the breeding season. Given that the sex determination of amphibians is temperature dependent, the male rate during embryonic development is positively correlated with ambient temperature [42, 43]. The breeding season of *B. orientalis* is later than that of other amphibians and, thus, the environmental temperature is also relatively higher; in addition, this species spawns in batches, so that egg hatching occurs throughout the breeding season [44]. In addition, batch spawning can also reduce the inbreeding caused by promiscuity.

## Conclusions

In conclusion, the northern population of *Bombina orientalis* is a typical naturally dispersed population, and has evolved effective adaptive strategies to maintain a stable population. The essence of the maintenance mechanism is to increase the genetic diversity through the long-distance biased dispersal behavior of males and the pattern of promiscuity. The relevance of these maintenance mechanisms for naturally dispersed populations provide a new perspective for further understanding the processes of speciation and evolution.

## Methods

### Sample collection and laboratory methods

We collected tissue samples of *B. orientalis* from 12 sites spanning the major geographical distribution range of this species (38° 43′–53° 33′ N, 118° 53′–135° 05′ E, Fig. 1A). The 515 samples, toes of the hind legs, were collected from different individuals in breeding grounds and nearby areas during the breeding season (May–July) in 2016–2018. Individuals wounds were disinfected with a pain relieving, antiseptic and antibacterial Bactine® spray before being released at the capture site. Samples were stored in per-labeled tubes filled with 95 % ethanol. All individuals were release back into the wild after tissue collection. We assigned sex to all 515 individuals before released. The Oriental fire-bellied toad is sexually dimorphic. In breeding adult males, their fore limbs are stout, inner metacarpal tubercles on the base of fingers I–III (female doesn’t have these tubercles), chest usually with scattered small black spines [9]. Sample sizes per locality are indicated in Additional file 1: Table S1. This study was approved by the Ethics and Experimental Animal Committee of Northeast Agricultural University, China.

DNA was extracted from the alcohol-preserved tissues following a standard proteinase K protocol [45]. The primers for the amplified mitochondrial genes *COI* and *Cytb* were as detailed in Zheng et al. [46]. Here *COI* was amplified using forward primer (5′-CAAATCACAAAGACATTGGCACCCT-3′) and reverse primer (5′-GATACGACATAGTGGAAGTGGGCTAC-3′). *Cytb* was amplified using forward primer (5′-GTATGTCACCCAACCTCCGAAAATC-3′) and reverse primer (5′-CAACTGGTTGTCCTCCAATTCATG-3’). PCR was conducted using 25-µl reaction systems including: 20 ng of template DNA, 0.2 µl Taq DNA polymerase (TaKaRa Taq™, 5 U/µl), 1 µl MgCl_2_ (TaKaRa Taq™, 25 mM), 1 µl dNTP Mixture (TaKaRa Taq™, 2.5 mM each), and 0.80 µl of each primer (10 µM) in 2.5 µl 10×PCR buffer (TaKaRa Taq™). Each PCR was conducted using the following parameters: initial denaturation at 95 °C for 5 min, followed by 30 cycles of denaturation at 95 °C for 1 min, annealing at 56 °C for 1 min, an extension at 72 °C for 1 min, and a final additional extension step at 72 °C for 10 min. Raw sequences were compared visually to the original chromatograms to avoid reading errors. We randomly selected 10–15 individuals from each sampling location for *COI* and *Cytb* mitochondrial gene acquisition. 131 samples from the northern population were successfully amplified. The other 127 mitochondrial sequences from the Korean peninsula were obtained from Fong et al. [10]. All 258 sequences were aligned using BioEdit 7.0.9 [47]. We also genotyped 515 individuals at 12 polymorphic loci following the methods described in Shi et al. [13].

### Population genetics analyses

The number of haplotypes (*H*), the nucleotide diversity index (*π*), and the haplotype diversity (*Hd*) were used to estimate the genetic diversity of mitochondrial *COI* and *Cytb* using DnaSP v. 5.10 [48]. Inbreeding coefficients (*F*_*IS*_) and genetic differences among population (*φ*_*ST*_) were provided in the program package Arlequin v. 3.5 [49].

Tests for linkage disequilibrium (LD) and Hardy–Weinberg equilibrium (HWE) were performed based on 12 microsatellite loci using GENEPOP v. 4.2 [50]. Default Markov chain parameters were used for both analyses. Based on these microsatellite loci, which conformed to HWE, the nuclear DNA diversity was calculated using GenAlEx 6.5 [51], including the number of alleles (*Na*), the effective number of alleles (*Ne*), observed heterozygosity (*Ho*), and expected heterozygosity (*He*).

### Mantel test

An isolation-by-distance (IBD) test was conducted by examining the correlation between Rousset’s *F*_*ST*_/(1-*F*_*ST*_) and the geographic distance [52]. All northern populations were analyzed using seven loci that conformed to HWE. All geographic distances were calculated using an online coordinate distance calculator (http://boulter.com/gps/distance/). A Mantel test with 10,000 permutations was conducted using GENEPOP [50].

### Population history analysis

Approximate Bayesian computation (ABC) as implemented in the program DIYABC was used to explore the genetic history of the northern population of *B. orientalis* and to determine changes in its effective population size (*Nan* and *N*) [53, 54]. The 12 northern populations were divided into two geographic groups (CH1 ~ CH3 located in lateral range of the Changbai mountains and CH4 ~ CL12 in the main range of the Changbai mountains) to form a simple scenario for preliminary investigations. This step was used to determine the parameters of the operation. Then, three scenarios corresponding to the classic dispersal history were constructed. In order to explore the regional dispersal history between the peninsula population and the northern population, we used mitochondrial data of three populations to construct the scenarios. According to Fong et al. we divided the peninsula population into the southern group (population 1) and northern group (population 3), population 2 represented the northern population in China. We tested whether the northern Korean group (pop 3) was derived from the northern population in China (pop 2) in scenario 1, or the other way around (scenario 2) or whether the northern population in China (pop 2) originated from the southern Korean group (pop 1) in scenario 3. For demographic analyses and local dispersal history within the northern population, we used mitochondrial data and microsatellite loci of three populations to construct the scenarios. Population 1 included CJ9–CJ11 and CL12, while population 2 included CH4–CH8, and population 3 included CH1–CH3. We tested whether pop 3 (CH1-CH3) originated from pop 2 (CH4-CH8) in scenario 1, or the other way around (scenario 2) or whether pop 2 originated from pop 1 (CJ9-CJ11 and CL12) in scenario 3. Default minimum and maximum priors (10 ~ 10,000) were used for all parameters. Conditions were placed on splitting time points so that t2 ≥ t1. Default priors were used for the mutation model. Three summary statistics were used in the Bayesian analysis for mitochondrial data, including Mean of numbers of the rarest nucleotide at segregating sites, Variance of numbers of the rare nucleotide at segregating sites and *Fst* [55]. Ten summary statistics were used in the Bayesian analysis for microsatellite loci, including mean number of alleles across loci, mean gene diversity across loci [56], mean allele size variance across loci, mean M index across loci [57, 58], mean number of alleles across loci (two samples), mean gene diversity across loci (two samples), mean allele size variance across loci (two samples), *F*_*ST*_ between two samples [59], shared allele distance between two samples [60] and (δµ) distance between two samples [61]. Model checking was performed using a principal component analysis (PCA) and posterior probabilities of each scenario were then calculated using a logistic regression of 1 % of the simulated data. The scenario(s) with the highest posterior probability were selected from each group. These scenarios were then compared in a final run, simulating 10^6^ data sets per scenario.

### Ecological niche modeling

To predict the geographic distribution of suitable habitat for *B. orientalis* in three time-scales recently (1960–1990 s), during the Middle Holocene (6000 years ago), and the LGM (22,000 years ago), ecological niche modeling (ENM) was conducted with Maxent v. 3.3.3 [62]. The records of *B. orientalis* occurrence were collected from field research during 2016 to 2018 and from the study by Fong et al. [10]. In addition, 19 bioclimatic variables were obtained from the WorldClim database (http://www.worldclim.org) [63] with resolutions of 2.5 arc-minutes as the environmental layer. Maxent analyses were run with 75 % of the species records for training and 25 % for testing the model. Bootstrapping was selected as the replicated run type and the number of replicates was set to 10, whereas the other parameters were kept as defaults.

### Tests for sex-biased dispersal

Three genetic methods were used to detect potential sex-biased dispersal: (1) comparison of the corrected assignment index (including mean *AIc*/*mAIc* and variance *AIc*/*vAIc*) between the sexes; (2) estimation of first-generation migrants; and (3) indirect genetic methods.

The corrected assignment index (*mAIc* and *vAIc*) was used to assess sex-biased dispersal using GenAlEx 6.5 [51]. *AIc* of an individual k sampled in population I is the probability that its genotype occurred by chance in population I. Thus, the more dispersed sex would have a lower *mAIc* or a larger *vAIc* than the philopatric sex. To calculate *AIc* for each sex, we only used the populations that the individual number per sex is more than 3, including CH1, CH2, CH3, CH6, CH7, CJ9, and CJ11 populations, and the total northern population.

First-generation migrants were detected for each sex using the program GENECLASS2 v. 2.0 [64]. First-generation migrants were defined as individuals that were born at a site other than the one in which they were collected. *Lh* (L_home, the likelihood of the individual genotype within the population where the individual was sampled) was used as the statistical criterion for likelihood computation [65]. To determine the critical value of the test statistic at α = 0.01 level, the Bayesian method of Rannala and Mountain was used in combination with the Monte Carlo resampling algorithm of Paetkau et al. [65]. The source sites of each migrant were identified according to their assignment results. Only loci conforming to HWE were used in the analysis.

In addition, *F*_*ST*_ was also used to measure the difference in the genetic differentiation between females and males. Trochet et al. (2016) reported that dispersal is assumed to be biased toward the gender with the lower *F*_*ST*_ [66]. Given that nuclear DNA is inherited from both parents and mtDNA is inherited from the maternal lineage only, male-biased dispersal was assumed when the mtDNA differentiation between male and female was higher than that of the nuclear DNA.

### Mating system analyses

The mating system of *B. orientalis* was comprehensively verified by determining the sex ratio and inbreeding coefficients (*F*_*IS*_) in each population using GENEPOP v. 4.0.10 [50], and the parentage analysis of individuals in the breeding grounds. Parentage analysis was conducted on samples collected from 2016 to 2018 in the CH3 breeding grounds by using Cervus v. 3.0 [67] to determine the relationship between individuals. To avoid subjective errors, all females collected in any one year at the breeding grounds were assumed to be candidate mothers and all individuals were assumed to be candidate offspring for maternity analysis. Then, identified offspring samples were included as candidate offspring and all males in the same year at breeding grounds were included as candidate fathers for paternity analysis. The mating system of *B. orientalis* was revealed from the corresponding parents of the same offspring. The higher and more significant the *LOD* values, the more likely there was to be linear relatedness.

## Supplementary Information

**Additional file 1.** Additional figures and tables.

## Data Availability

All newly acquired sequences have been deposited in GenBank® repository (http://www.ncbi.nlm.nih.gov) under accession numbers MK609566-MK609843 (see Additional file 1: Table S1). Microsatellite DNA data has uploaded as online supporting information (https://datadryad.org/stash/share/dSXduyrTUZnDZeg6hOO7oN3S4bigmmAxZbk_WF2jRh8).

## Abbreviations

*COI*: Mitochondrial cytochrome c oxidase subunit I gene
*Cytb*: Mitochondrial cytochrome b gene
*H*: The number of haplotypes
*Hd*: The haplotype diversity
*π*: The nucleotide diversity index
LD: Linkage disequilibrium
HWE: Hardy–Weinberg equilibrium
*Nan*: effective population sizes of the ancestral
*N*: effective population sizes of contemporary
*Na*: The number of alleles
*Ne*: The effective number of alleles
*He*: Expected heterozygosity
*Ho*: Observed heterozygosity
IBD: Isolation-by-distance
LGM: Last Glacial Maximum
*mAIc*: The corrected assignment index
*F*_*ST*_: Genetic differences among population
*F*_IT_: total-population inbreeding coefficients
*F*_*IS*_: Inbreeding coefficients
ENM: Ecological niche modeling
PCR: Polymerase chain reaction
bp: Base pairs
ABC: Approximate Bayesian computation
PCA: Principal component analysis
*LOD*: The log-likelihood ratio for a parent-offspring relationship between the known parent and the offspring
