# Supplementary material for: Dispersal and mating patterns determine the fate of naturally dispersed populations: evidence from Bombina orientalis
Source: BMC Ecol Evol. 2021 Jun 7;21:111. doi: 10.1186/s12862-021-01844-3 (PMC8182911; doi:10.1186/s12862-021-01844-3)

**Supplemental Information for:**

**Dispersal and mating patterns determine the fate of naturally dispersed population: Evidence from *Bombina orientalis***

**Liqun Yu^1^, Shuai Zhao^1^, Fanbing Meng^1^, Yanshuang Shi^1^, Chunzhu Xu^1^**

^1^ College of Life Science, Northeast Agricultural University, Harbin, China

**Appendix**

Supplementary Table 1. Locality information, sample information, and Genbank accession number of specimens. Sample size: numbers in < > are sample sizes subjected to mtDNA sequences; the other numbers are sample sizes of microsatellite DNA.

Supplementary Table 2. The P-value of Hardy-Weinberg equilibrium at each population each locu.

Supplementary Table 3. The genetic diversity of each sampling site.

Supplementary Table 4. The prior parameters distribution for ABC analyses and the posterior parameters estimation for scenario 1 (see Figure 2 for scenario topography). N, effective population size; Nan, effective population size of a common ancestor population. The sample locations for each analysis are given in parentheses.

Supplementary Table 5. Identified first-generation migrants. Including sex of migrants, and direct geographical distance from migrants’ place of origin to sampling sites.

Supplementary Table 6. The ratio of males to females in each sampling sites. All means the entire population.

Supplementary Table 7. The identified offspring in CH3 breeding ground and their most likely parents ID.

Supplementary Figure 1. Results from isolation by distance analysis of microsatellite loci with a Mantel test. *P*_Mantel_ < 0.0001, *r* = 0.510.

Supplementary Figure 2. Results of species distribution modeling in *B.orientalis* based on 19 bioclimatic variables (The blue colored 1 are not suitable for species; the light blue colored 2 are a little suitable for species; the yellow colored 3 are suitable for species; the red colored 4 are very suitable for species)

Supplementary Table 1

| Location name | Abbreviation | Coordinates | Sample number | Genbank accession number | Sample sizes |
| --- | --- | --- | --- | --- | --- |
| Chenjia Village, Heilongjiang, China | CH1 | 127.56°E, 44.89°N | BOC039~041, BOC043~045, BOC049~051, BOC053, BOC056, BOC061, BOC062, BOC069~098, BOC106~113, BOC141~147, BOC149, BOC159~187 | MK609566~MK609568, MK609570~MK609579, MK609634, MK609635, MK609705~MK609707, MK609709~MK609718, MK609773, MK609774 | <15>  87 |
| Xiaoling, Heilongjiang, China | CH2 | 127.18°E, 45.21°N | BOC188, BOC189, BOC192~200, BOC203~205, BOC207, BOC209~219, BOC221~229, BOC231, BOC232, BOC235, BOC237~255 | MK609596~MK609605, MK609640~MK609643, MK609735~MK609744, MK609779~MK609782 | <14>  58 |
| Maoer Mountain, Heilongjiang, China | CH3 | 127.50°E, 45.27°N | BOC001~015, BOC017~035, BOC100~102, BOC475, BOC477~479, BOC481~484, BOC487~491, BOC494~500, BOC502~524, BOC526~529, BOC531, BOC534, BOC535, BOC537~539, BOC541, BOC542, BOC544~548, BOC550, BOC604~637 | MK609580~MK609587,  MK609636, MK609637,  MK609719~MK609726, MK609775, MK609776 | <10>  132 |
| Weihe, Heilongjiang, China | CH4 | 128.21°E, 44.49°N | BOC297~333 | MK609606~MK609609, MK609653~MK609657, MK609745~MK609748, MK609792~MK609796 | <9>  37 |
| Weihu Mountain, Heilongjiang, China | CH5 | 129.29°E, 44.81°N | BOC066~068, BOC256~269 | MK609588~MK609595, MK609638, MK609639, MK609727~MK609734, MK609777, MK609778 | <10>  17 |
| Sandaoguan, Heilongjiang, China | CH6 | 129.31°E, 44.46°N | BOC430~458, BOC463~470 | MK609617~MK609620, MK609645~MK609649, MK609685, MK609686, MK609688, MK609756~MK609759, MK609784~MK609788, MK609824, MK609825, MK609827 | <12>  37 |
| Mudanfeng, Heilongjiang, China | CH7 | 129.43°E, 44.29°N | BOC393~417, BOC421~426, BOC428, BOC429 | MK609663~MK609668, MK609670~MK609676, MK609683, MK609684, MK609802~Mk609807, MK609809~MK609815, MK609822, MK609823 | <15>  33 |
| Dingning, Heilongjiang, China | CH8 | 131.07°E, 44.10°N | BOC593~603 | MK609677~MK609682, MK609799~MK609704, MK609816~MK609821, MK609839~MK609843 | <11>  11 |
| Lushui River, Jilin, China | CJ9 | 127.84°E, 42.59°N | BOC335~364, BOC367~369, BOC371~378, BOC381, BOC383~388, BOC390~392, BOC585~592 | MK609610~MK609616, MK609658~MK609662, MK609749~MK609755, MK609797~MK609801 | <12>  59 |
| Linjiang, Jilin, China | CJ10 | 126.91°E, 41.81°N | BOC117~122  (Unknow sex) | MK609630~MK609633, MK609652, MK609699, MK609769~MK609772, MK609791, MK609838 | <6>  6 |
| Yulin Town, Liaoning, China | CJ11 | 125.50°E, 41.03°N | BOC552~560, BOC562~584 | MK609621, MK609622, MK609650, MK609651, MK609687, MK609689~ MK609693, MK609760, MK609761, MK609789, MK609790, MK609826, MK609828~ MK609832 | <11>  32 |
| Kuandian, Heilongjiang, China | CL12 | 124.78°E, 40.73°N | BOC116, BOC128~132  (Unknow sex) | MK609694~ MK609698, MK609833~ MK609837 | <5>  6 |
| Korean peninsula | - | 126.38°~129.23°E, 33.25°~38.37°N | BO001, BO002, BO010, BO011, BO025, BO031~BO038, BO055, BO056, BO060~BO065, BO075, BO076, BO114~ BO120, BO125, BO126, BO192, BO193, BO219, BO235, BO237, BO317, BO318, BO701, BO702, BO705, BO706, BO710, BO711, BO713~BO715, BO717~BO720, BO722, BO729~BO732, BO734~ BO736, BO748~BO752, BO754~BO756, BO761, BO764~BO767, mms0594, mms0665~mms0667, mms0878~mms0882, mms0931, mms0936, mms0943, mms0945, mms0946, mms0952~mms0954, mms1392, mms1393, mms1435, mms1437, mms1469, mms1470, mms2036~ mms2038, mms2074, mms2120, mms2121, mms2124, mms2138, mms2139, mms3038, mms3039, mms3159, mms3187, mms3228, mms3229, mms3252, mms3263, mms3539, mms3540, mms5848, mms5849, mms5930, mms5990, mms5991, mms6030, mms6031, mms6280, mms6283, mms6284, mms6285, mms6286 | KR869225~KR869512 | <127> |

Supplementary Table 2

|  | CH1 | CH2 | CH3 | CH4 | CH5 | CH6 | CH7 | CH8 | CJ9 | CJ10 | CJ11 | CL12 |
| --- | --- | --- | --- | --- | --- | --- | --- | --- | --- | --- | --- | --- |
| 9H | 0.2016 | 0.7119 | 0.0472 | 0.0245 | 0.7908 | 0.0169 | 0 | 0.0016 | 0.9035 | 0.0079 | 0.0005 | 0.1571 |
| 12F | 0.6485 | 0.0403 | 0.0177 | 0.4846 | 0.3358 | 0.8656 | 0.3880 | 0.3468 | 0.3462 | 0.0768 | 0.0008 | 0.7780 |
| 13 | 1 | 1 | 0.1482 | 0.6413 | 0.9727 | 0.8533 | 0.1383 | 0.4812 | 0.3279 | - | 0.5400 | 0.0611 |
| 17 | 0.1001 | 0.0558 | 0.1595 | 0.6197 | 0.6613 | 0.0045 | 0.4613 | 0.0254 | 0.1482 | 0.1638 | 0.0562 | 0.2021 |
| 19 | 0 | 0.0769 | 0 | 0 | 0.0001 | 0.0294 | 0 | 0.0669 | 0 | 0.0150 | 0.0001 | 0.0839 |
| 141 | 0 | - | 1 | - | - | 0 | 0 | - | 0 | 0.0211 | 0 | 0.0904 |
| 10F | 0.3036 | 0.8403 | 0.0177 | 0.7819 | 1 | 0.5631 | 0.0704 | 0.3524 | 0.1014 | 0.0848 | 0.0079 | 0.4827 |
| 23 | 0.0058 | 0.0021 | 0 | 0 | 0.1012 | 0 | 0.5904 | 0.2434 | 0.0027 | 0.3435 | 0.0001 | 0.0920 |
| 105 | 0 | 0 | 0.0001 | 0.0003 | 0.2214 | 0.0426 | 0.2430 | 1 | 0.0108 | 0.0909 | 1 | 1 |
| 42 | - | 1 | 0.0108 | 1 | 1 | 0.0914 | 0.5116 | 0.6250 | 0.2619 | - | 0.0018 | 0.0490 |
| 53 | 0.7896 | 0.0019 | 0.9030 | 0.9961 | 0.1488 | 0.0032 | 0.0266 | 0.7453 | 0.7154 | 0.7226 | 0.1702 | 0.0307 |
| B14 | 0.0054 | 0.4921 | 0 | 0.9059 | 0.2247 | 0.3490 | 0.0221 | 0.2840 | 0.5116 | 0.4253 | 0.3311 | 0.2887 |

Supplementary Table 3

| Pop | *Na* | *Ne* | *Ho* | *He* |
| --- | --- | --- | --- | --- |
| CH1 | 3.714±1.017 | 2.207±0.453 | 0.399±0.112 | 0.423±0.113 |
| CH2 | 4.286±0.865 | 2.310±0.415 | 0.467±0.097 | 0.470±0.099 |
| CH3 | 5.286±1.149 | 2.460±0.521 | 0.447±0.096 | 0.481±0.101 |
| CH4 | 5.714±1.523 | 3.421±0.727 | 0.674±0.085 | 0.620±0.077 |
| CH5 | 4.571±0.948 | 3.188±0.618 | 0.603±0.088 | 0.593±0.088 |
| CH6 | 5.571±1.152 | 2.814±0.348 | 0.571±0.067 | 0.606±0.056 |
| CH7 | 6.857±1.455 | 4.322±0.741 | 0.706±0.052 | 0.734±0.036 |
| CH8 | 4.857±0.634 | 3.007±0.310 | 0.662±0.033 | 0.647±0.034 |
| CJ9 | 6.571±1.645 | 3.510±1.125 | 0.595±0.076 | 0.588±0.077 |
| CJ10 | 3.429±0.429 | 2.529±0.409 | 0.476±0.099 | 0.516±0.096 |
| CJ11 | 7.000±0.976 | 3.323±0.675 | 0.606±0.082 | 0.629±0.060 |
| CL12 | 3.571±0.429 | 3.064±0.426 | 0.676±0.122 | 0.635±0.047 |
| ALL | 5.036±0.327 | 2.961±0.176 | 0.573±0.027 | 0.568±0.024 |

Supplementary Table 4

| Parameter | Median | Quantile (2.5%) | Quantile (97.5%) |
| --- | --- | --- | --- |
| Scenario 1 (within the northern population) | | | |
| *N*1_(CJ9~CJ11, CL12)_ | 8460 | 5140 | 9900 |
| *N*2_(CH4~CJ8)_ | 5570 | 3320 | 9760 |
| *N*3_(CH1~CH3)_ | 3910 | 1740 | 9640 |
| *Nan* | 2450 | 1130 | 8770 |
| µmic | 4.53 × 10^-4^ | 2.04 × 10^-4^ | 8.70 × 10^-4^ |

Supplementary Table 5

| Sample number | Sex | Sampling Sites | Place of origin | Geographical distance |
| --- | --- | --- | --- | --- |
| BOC012 | ♂ | CH3 | CH2 | 24.7 km |
| BOC458 | ♂ | CH6 | CH5 | 35.9 km |
| BOC479 | ♂ | CH3 | CH1 | 40.4 km |
| BOC049 | ♂ | CH4 | CH1 | 69.0 km |
| BOC432 | ♂ | CH6 | CH2 | 183.3 km |
| BOC374 | ♂ | CH9 | CH7 | 229.8 km |
| BOC616 | ♂ | CH3 | CJ11 | 478.0 km |
| BOC396 | ♂ | CH7 | CJ11 | 480.8 km |
| BOC626 | ♂ | CH3 | CH3 | — |
| BOC322 | ♂ | CH4 | CH4 | — |
| BOC454 | ♀ | CH6 | CH5 | 35.9 km |
| BOC421 | ♀ | CH4 | CH4 | — |

Supplementary Table 6

|  | Sampling time | Number of male(♂) | Number of female(♀) | Male/Female |
| --- | --- | --- | --- | --- |
| CH1 | 2016.07 | 48 | 2 | 68/19 |
|  | 2017.05 | 20 | 17 |  |
| CH2 | 2017.06 | 46 | 12 | 19/4 |
| CH3 | 2016.07 | 33 | 4 | 68/27 |
|  | 2017.07 | 45 | 16 |  |
|  | 2018.07 | 23 | 11 |  |
| CH4 | 2017.06 | 36 | 1 | 36/1 |
| CH5 | 2017.06 | 16 | 1 | 16/1 |
| CH6 | 2017.06 | 24 | 13 | 24/13 |
| CH7 | 2017.06 | 26 | 7 | 26/7 |
| CH8 | 2018.06 | 11 | 0 | 11/0 |
| CJ9 | 2017.06 | 52 | 7 | 52/7 |
| CJ11 | 2017.06 | 20 | 12 | 5/3 |
| ALL | — | 400 | 103 | 400/103 |

Supplementary Table 7

| Sampling time | Offspring ID | Most likely parents | |
| --- | --- | --- | --- |
|  |  | Mother ID | Father ID |
| CH3-2016 | BOC015 | BOC004 | BOC020 |
|  | BOC018 |  | BOC030 |
|  | BOC011 | BOC013 | BOC030 |
| CH3-2017 | BOC538 | BOC512 | BOC500 |
|  | BOC484 |  | BOC500 |
|  | BOC477 |  | BOC534 |
|  | BOC496 |  | BOC490 |
|  | BOC478 | BOC550 | BOC529 |
|  | BOC537 |  | BOC529 |
|  | BOC488 | BOC548 | BOC498 |
|  | BOC531 | BOC516 | BOC498 |
|  | BOC528 |  | BOC524 |
|  | BOC509 | BOC517 | BOC500 |
|  | BOC519 |  | BOC534 |
| CH3-2018 | BOC614 | BOC628 | BOC612 |
|  | BOC617 | BOC635 | BOC612 |
|  | BOC623 |  | BOC620 |

Supplementary Figure 1


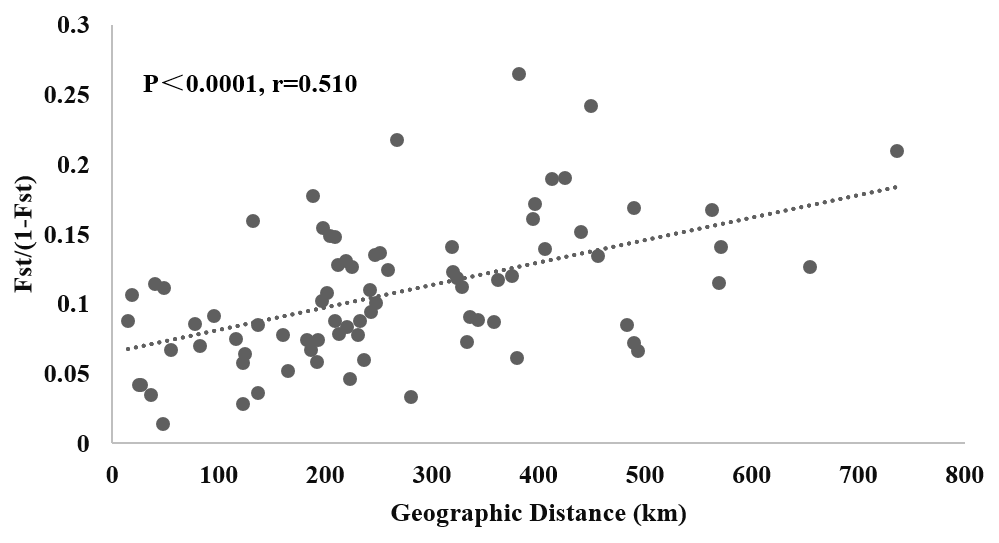


Supplementary Figure 2


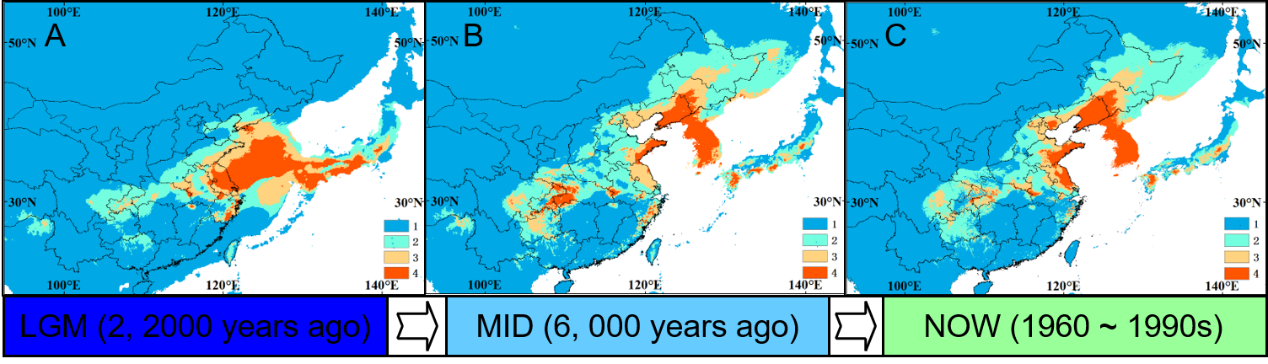

Supplement: Supplementary file 1 — Additional file 1. Additional figures and tables. [file 12862_2021_1844_MOESM1_ESM.doc]
